# Supplementary material for: Systematic Mendelian randomization using the human plasma proteome to discover potential therapeutic targets for stroke
Source: Nat Commun. 2022 Oct 17;13:6143. doi: 10.1038/s41467-022-33675-1 (PMC9576777; doi:10.1038/s41467-022-33675-1)
Supplement: Supplementary file 1 — Supplementary Information [file 41467_2022_33675_MOESM1_ESM.pdf]

# Discovery of potential therapeutic targets for stroke using human plasma proteome through systematic Mendelian randomization

**Supplementary Figures**

**Supplementary Figure 1.** Standard MR plots for proteins and risk of stroke. ......................................2
**Supplementary Figure 2.** Colocalization plots of pQTLs and genetic associations of stroke outcomes and risk factor(s). ........................................................................................................................ 10
**Supplementary Figure 3.** Standard MR plots for risk factors and stroke. ......................................... 11
**Supplementary Figure 4.** Standard MR plots for proteins and stroke risk factors............................. 12
**Supplementary Figure 5.** Manhattan plots of the potential on-target side-effects from Phe-MR analysis. ........................................................................................................................................ 14
**Supplementary Figure 6.** Forest plots illustrating the potential on-target side-effects associated with causal proteins revealed by Phe-MR analysis. ...................................................................... 16
**Supplementary Figure 7.** Correlations of causal effect sizes of six proteins on stroke outcomes with IVs derived from primary method and others....................................................................... 17

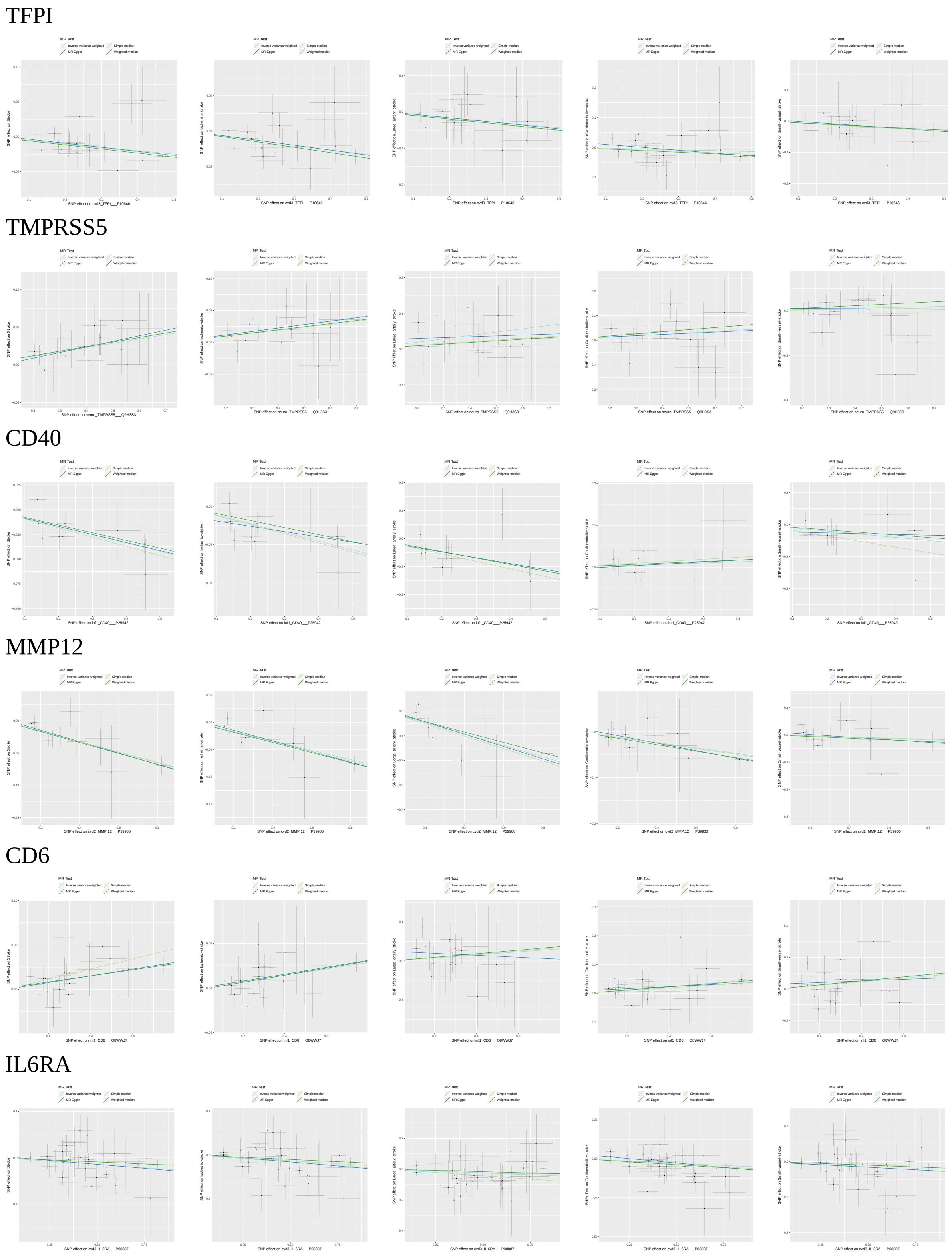


**Supplementary Figure 1.** Standard MR plots for proteins and risk of stroke.

Scatter plot showing the estimated effect sizes and 95% confidence intervals on plasma proteins (from linear regression; horizontal lines) and risk of five stroke outcomes (from logistic regression; vertical lines) for each variant used in the MR analyses.  The different regression lines indicate the effect sizes as calculated by different MR tests (Methods). Abbreviations: CD40 = B Cell Surface Antigen CD40;

TFPI = Tissue Factor Pathway Inhibitor; MMP12 = Matrix Metallopeptidase 12; IL6RA = Interleukin 6 Receptor Subunit Alpha; TMPRSS5= Transmembrane Serine Protease 5; CD6 = T-Cell Differentiation Antigen CD6.

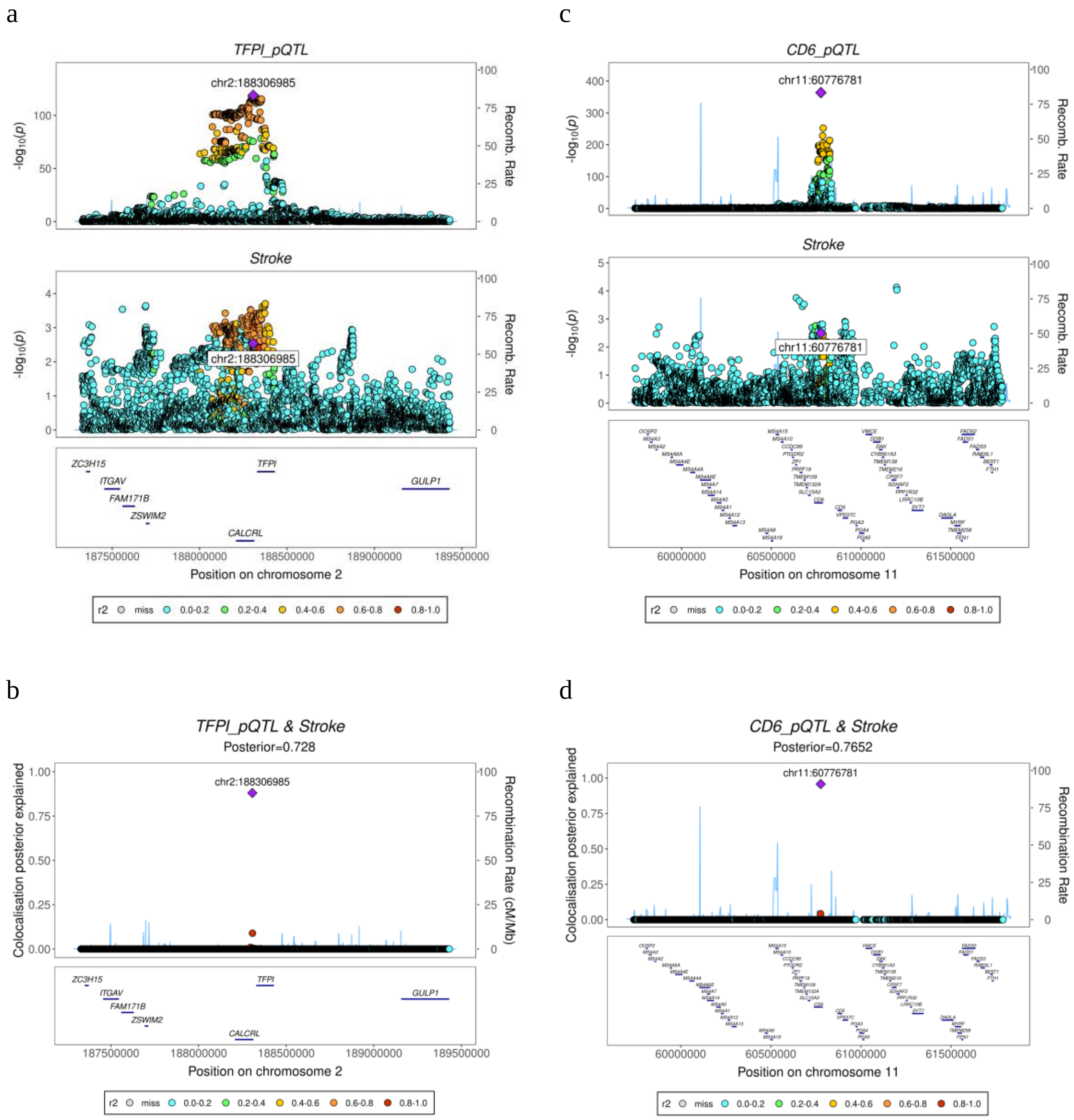
a
TFPI_pQTL
chr2:188306985
Stroke
chr2:188306985
-log10(p)
Recomb. Rate
ZC3H15
ITGAV
FAM171B
ZSWIM2
TFPI
CALCRL
GULP1
Position on chromosome 2
r2 miss 0.0-0.2 0.2-0.4 0.4-0.6 0.6-0.8 0.8-1.0
b
TFPI_pQTL & Stroke
Posterior=0.728
chr2:188306985
Colocalisation posterior explained
Recombination Rate (cM/Mb)
Position on chromosome 2
c
CD6_pQTL
chr11:60776781
Stroke
chr11:60776781
Position on chromosome 11
d
CD6_pQTL & Stroke
Posterior=0.7652
chr11:60776781
Colocalisation posterior explained
Recombination Rate
Position on chromosome 11

e

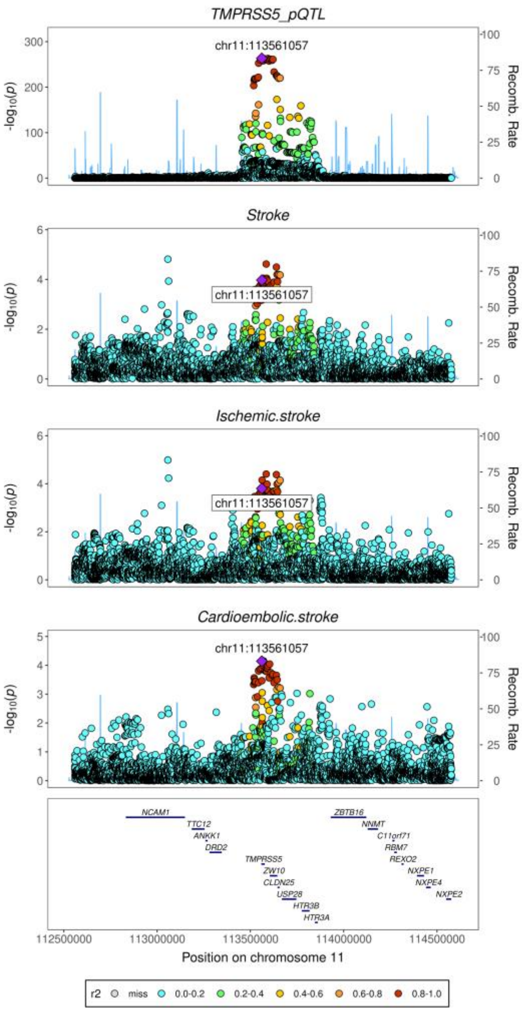
TMPRSS5_pQTL
chr11:113561057
Stroke
Ischemic.stroke
Cardioembolic.stroke
-log10(p)
Recomb. Rate
NCAM1
TTC12
ANKK1
DRD2
TMPRSS5
ZW10
CLDN25
USP28
HTR3B
HTR3A
ZBTB16
NNMT
C11orf71
RBM7
REXO2
NXPE1
NXPE4
NXPE2
112500000
113000000
113500000
114000000
114500000
Position on chromosome 11
r2 miss 0.0-0.2 0.2-0.4 0.4-0.6 0.6-0.8 0.8-1.0

g

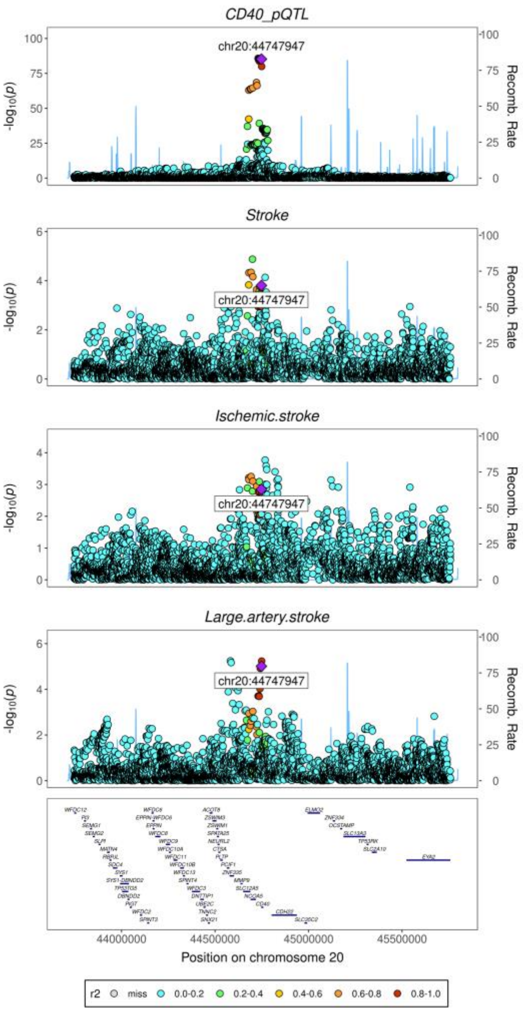
CD40_pQTL
chr20:44747947
Stroke
Ischemic.stroke
Large.artery.stroke
-log10(p)
Recomb. Rate
44000000
44500000
45000000
45500000
Position on chromosome 20
r2 miss 0.0-0.2 0.2-0.4 0.4-0.6 0.6-0.8 0.8-1.0

f

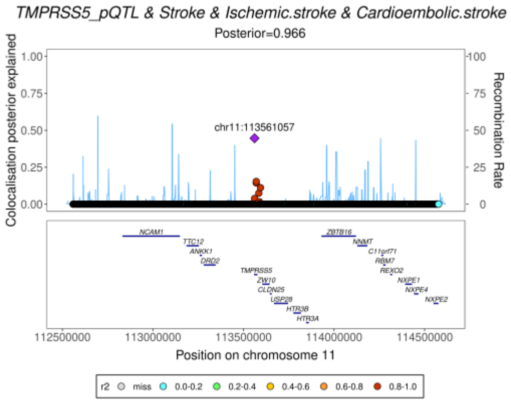
TMPRSS5_pQTL & Stroke & Ischemic.stroke & Cardioembolic.stroke
Posterior=0.966
chr11:113561057
Colocalisation posterior explained
Recombination Rate
112500000
113000000
113500000
114000000
114500000
Position on chromosome 11
r2 miss 0.0-0.2 0.2-0.4 0.4-0.6 0.6-0.8 0.8-1.0

h

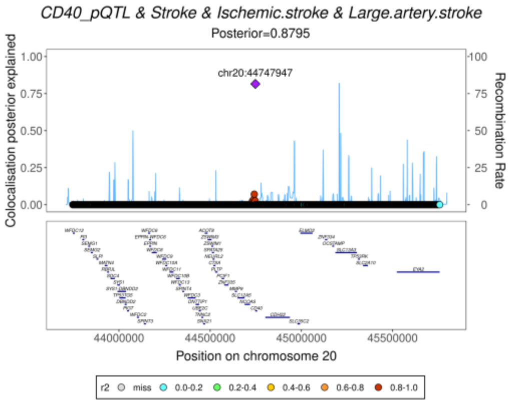
CD40_pQTL & Stroke & Ischemic.stroke & Large.artery.stroke
Posterior=0.8795
chr20:44747947
Colocalisation posterior explained
Recombination Rate
44000000
44500000
45000000
45500000
Position on chromosome 20
r2 miss 0.0-0.2 0.2-0.4 0.4-0.6 0.6-0.8 0.8-1.0

i

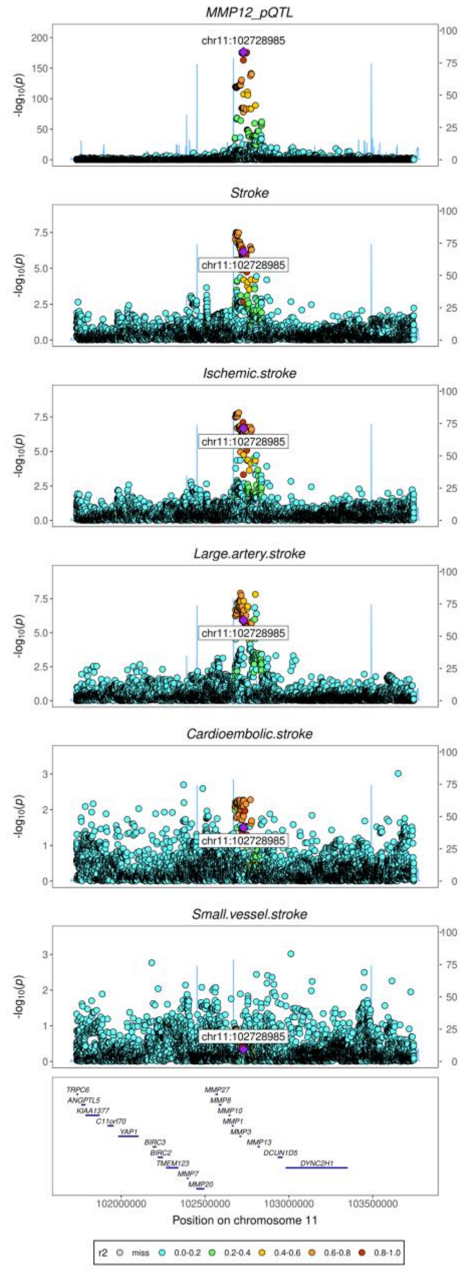
MMP12_pQTL
Stroke
Ischemic.stroke
Large.artery.stroke
Cardioembolic.stroke
Small.vessel.stroke
chr11:102728985
-log10(p)
Recomb. Rate
TRPC6
ANGPTL5
KIAA1377
C11orf70
YAP1
BIRC3
BIRC2
TMEM123
MMP7
MMP20
MMP27
MMP8
MMP10
MMP1
MMP3
MMP13
DCUN1D5
DYNC2H1
102000000
102500000
103000000
103500000
Position on chromosome 11
r2
miss
0.0-0.2
0.2-0.4
0.4-0.6
0.6-0.8
0.8-1.0

k

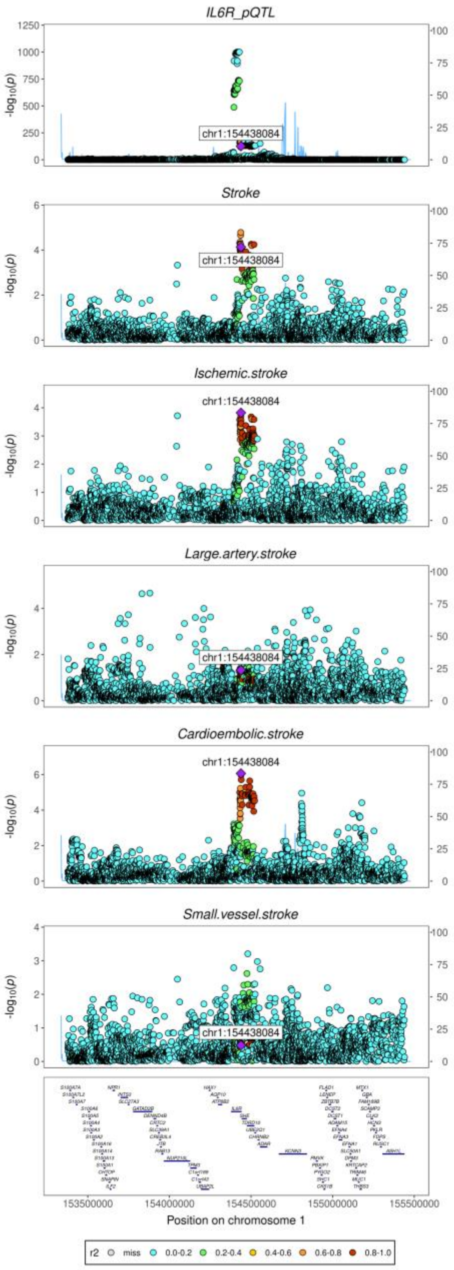
IL6R_pQTL
Stroke
Ischemic.stroke
Large.artery.stroke
Cardioembolic.stroke
Small.vessel.stroke
chr1:154438084
-log10(p)
Recomb. Rate
153500000
154000000
154500000
155000000
155500000
Position on chromosome 1
r2
miss
0.0-0.2
0.2-0.4
0.4-0.6
0.6-0.8
0.8-1.0

j

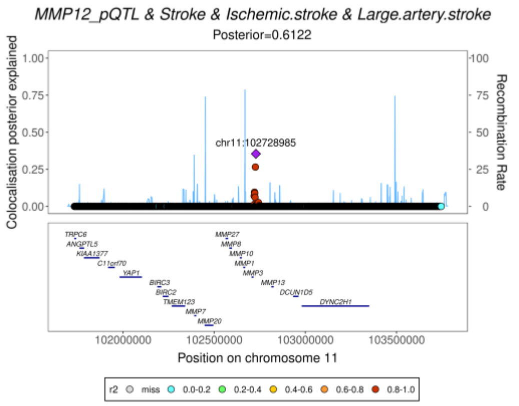
MMP12_pQTL & Stroke & Ischemic.stroke & Large.artery.stroke
Posterior=0.6122
chr11:102728985
Colocalisation posterior explained
Recombination Rate
102000000
102500000
103000000
103500000
Position on chromosome 11
r2
miss
0.0-0.2
0.2-0.4
0.4-0.6
0.6-0.8
0.8-1.0

l

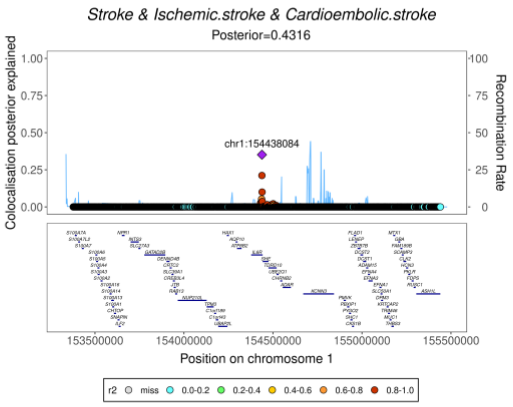
Stroke & Ischemic.stroke & Cardioembolic.stroke
Posterior=0.4316
chr1:154438084
Colocalisation posterior explained
Recombination Rate
153500000
154000000
154500000
155000000
155500000
Position on chromosome 1
r2
miss
0.0-0.2
0.2-0.4
0.4-0.6
0.6-0.8
0.8-1.0

m

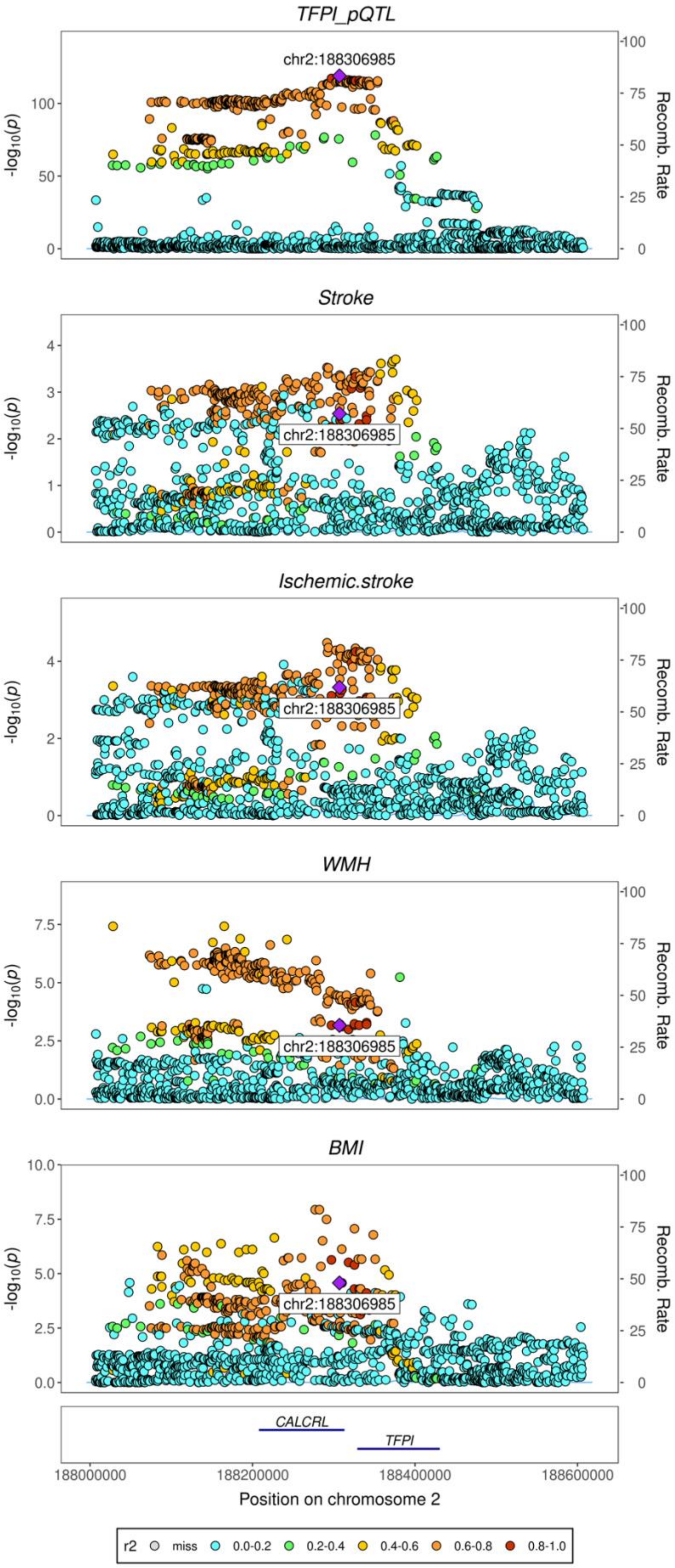
TFPI_pQTL
chr2:188306985
-log10(p)
Recomb. Rate
Stroke
Ischemic.stroke
WMH
BMI
CALCRL
TFPI
188000000
188200000
188400000
188600000
Position on chromosome 2
r2 miss 0.0-0.2 0.2-0.4 0.4-0.6 0.6-0.8 0.8-1.0

n

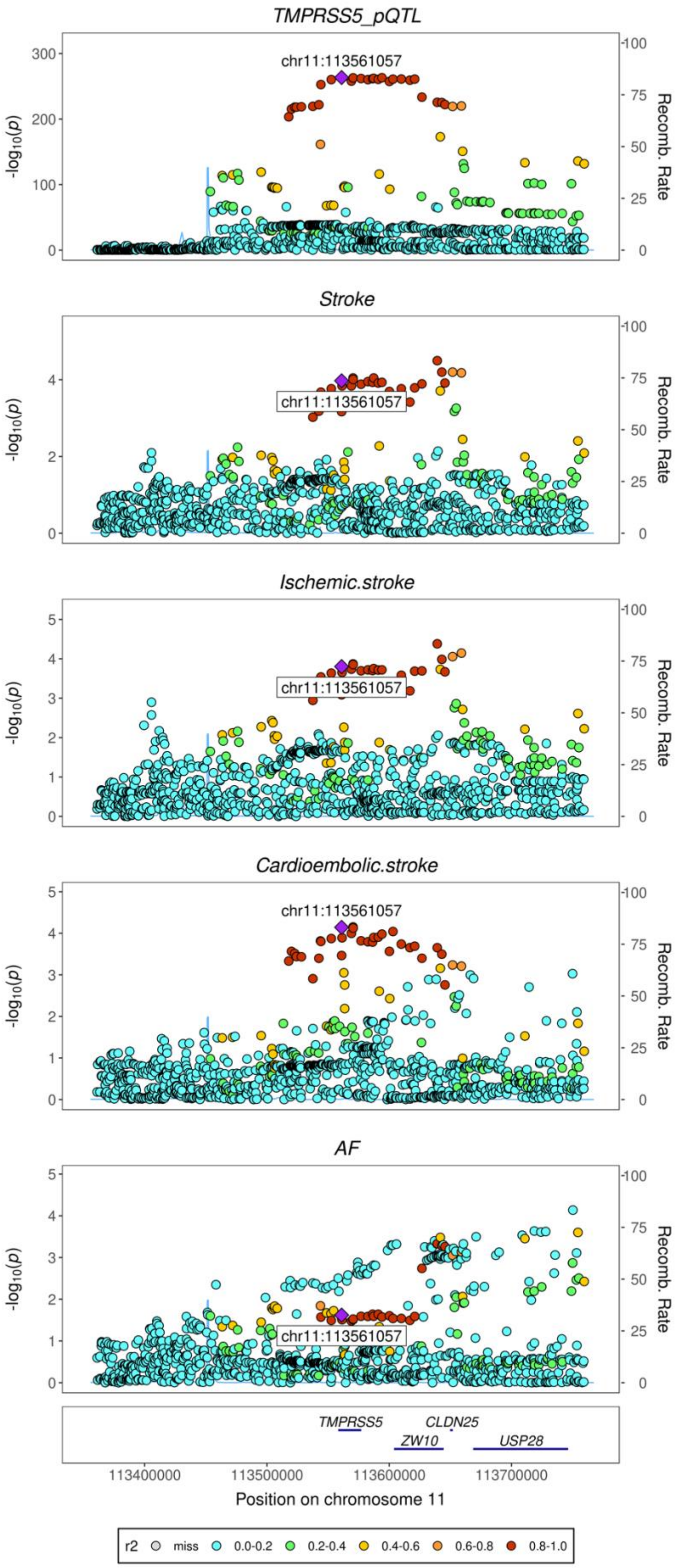

TMPRSS5_pQTL
chr11:113561057
Stroke
chr11:113561057
Ischemic.stroke
chr11:113561057
Cardioembolic.stroke
chr11:113561057
AF
chr11:113561057
-log10(p)
Recomb. Rate
TMPRSS5
CLDN25
ZW10
USP28
113400000
113500000
113600000
113700000
Position on chromosome 11
r2
miss
0.0-0.2
0.2-0.4
0.4-0.6
0.6-0.8
0.8-1.0

o

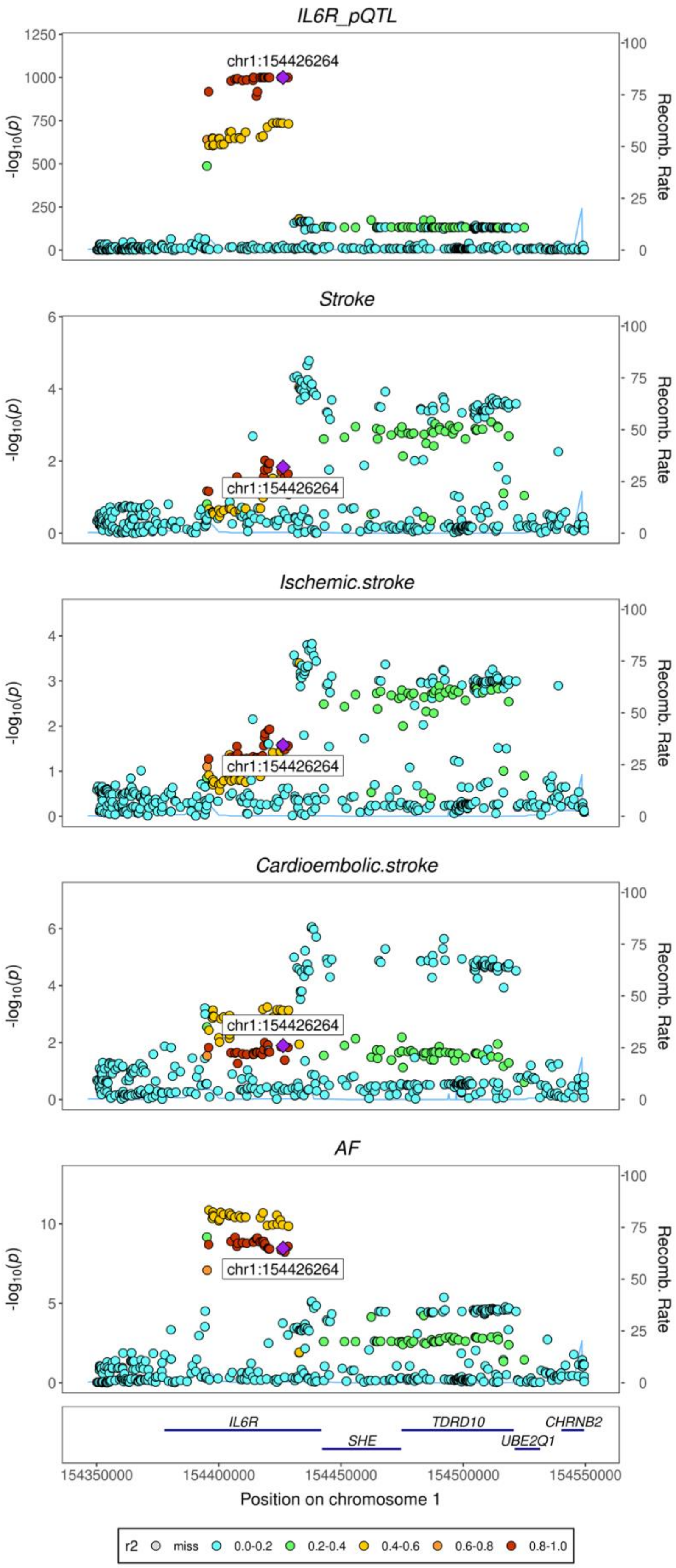

IL6R_pQTL
chr1:154426264
Stroke
chr1:154426264
Ischemic.stroke
chr1:154426264
Cardioembolic.stroke
chr1:154426264
AF
chr1:154426264
-log10(p)
Recomb. Rate
IL6R
SHE
TDRD10
UBE2Q1
CHRNB2
154350000
154400000
154450000
154500000
154550000
Position on chromosome 1
r2
miss
0.0-0.2
0.2-0.4
0.4-0.6
0.6-0.8
0.8-1.0

**Supplementary Figure 2.** Colocalization plots of pQTLs and genetic associations of stroke outcomes and risk factor(s).

a & b: colocalization of TFPI pQTLs and stroke association signals; c & d: colocalization of CD6 pQTLs and stroke association signals; e & f: colocalization of TMPRSS5 pQTLs and stroke association signals; g & h: colocalization of CD40 pQTLs and stroke association signals; i & j: colocalization of MMP12 pQTLs and stroke association signals; k & l: colocalization of IL6RA pQTLs and stroke association signals; m: stacked genetic association plots of TFPI pQTLs, stroke, ischemic-stroke, WHM and BMI associations; n: stacked genetic association plots of TMPRSS5 pQTLs, stroke, ischemic-stroke, cardioembolic-stroke and AF associations; o: stacked genetic association plots of IL6RA pQTLs, stroke, ischemic-stroke, cardioembolic-stroke and AF associations. a/c/e/g/i/k/m/n/o: regional association plot of pQTLs and stroke/risk factor associations. SNPs are plotted by their positions on the chromosome against association with the protein level and stroke/ risk factor ($-\log_{10} P$ value) on the left *y* axis. Points are coloured by their local linkage disequilibrium (LD) pattern with their "candidate causal SNP" (purple diamond). Blow the main plot is the track showing the position of local genes. b/d/f/h/j/l: plot of percentage of Posterior probability (PP) of colocalization explained by each of the SNP within the corresponding region. The traits that colocalized are presented in the title of the plot and the PP of colocalization is presented in the subtitle of the plot. SNPs are plotted by their positions on the chromosome against the percentage explained on the left *y* axis. The best candidate causal SNP is presented in purple diamond. Abbreviations: CD40 = B Cell Surface Antigen CD40; TFPI = Tissue Factor Pathway Inhibitor; MMP12 = Matrix Metallopeptidase 12; IL6RA = Interleukin 6 Receptor Subunit Alpha; TMPRSS5= Transmembrane Serine Protease 5; CD6 = T-Cell Differentiation Antigen CD6; AF = Atrial Fibrillation; WMH = White Matter Hyperintensity; BMI = Body Mass Index.

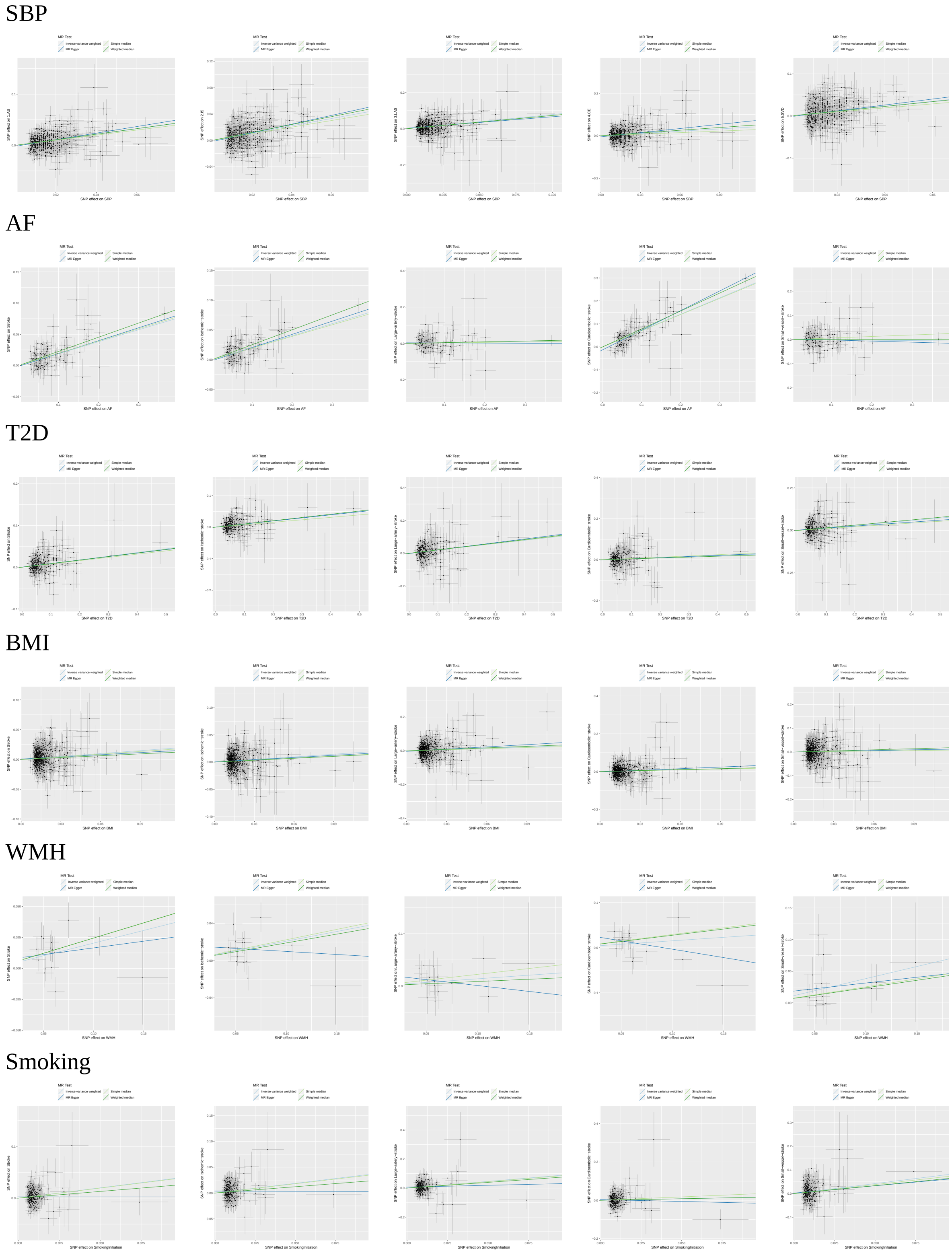


**Supplementary Figure 3.** Standard MR plots for risk factors and stroke.

Scatter plot showing the estimated effect sizes and 95% confidence intervals on risk factors and risk of five stroke outcomes for each variant used in the MR analyses. The different regression lines indicate the effect sizes as calculated by different MR tests (Methods). Abbreviations: SBP = Systolic Blood Pressure; AF = Atrial Fibrillation; WMH = White Matter Hyperintensity; T2D = Type 2 Diabetes; BMI = Body Mass Index; Smoking = Smoking Initiation.

TFPI and BMI

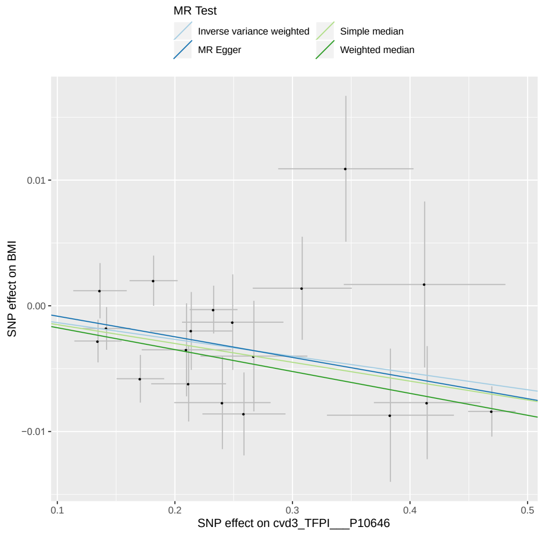


TFPI and WMH

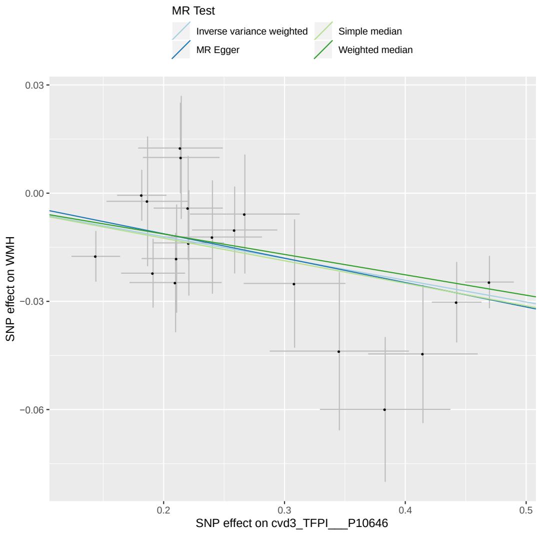


TMPRSS5 and AF

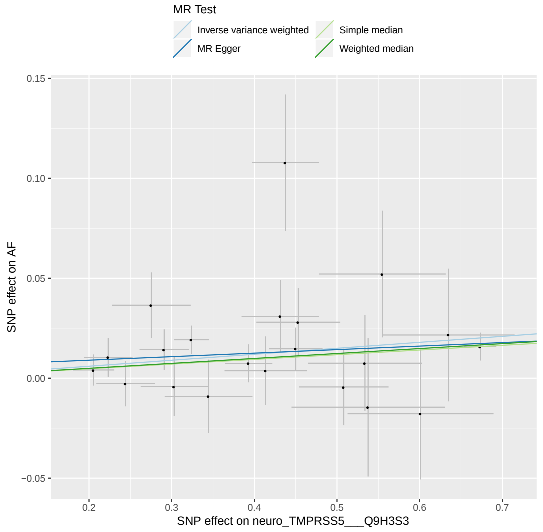


IL6RA and AF

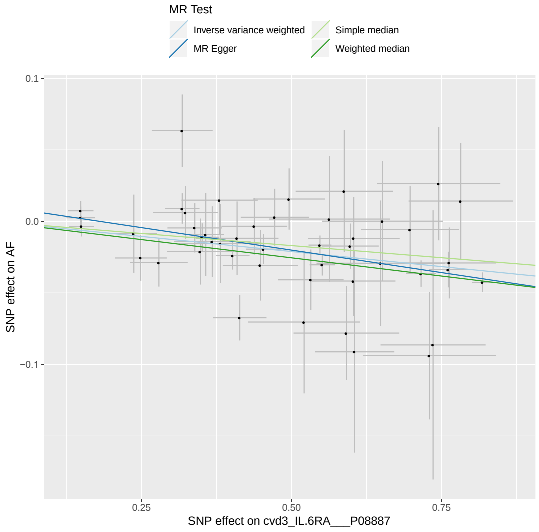


**Supplementary Figure 4.** Standard MR plots for proteins and stroke risk factors.

Scatter plot showing the estimated effect sizes and 95% confidence intervals on proteins and risk of stroke risk factors for each variant used in the MR analyses. The different regression lines indicate the effect sizes as calculated by different MR tests (Methods). Abbreviations: TFPI = Tissue Factor Pathway Inhibitor; IL6RA = Interleukin 6 Receptor Subunit Alpha; TMPRSS5= Transmembrane Serine Protease 5; AF = Atrial Fibrillation; WMH = White Matter Hyperintensity; BMI = Body Mass Index.

TFPI

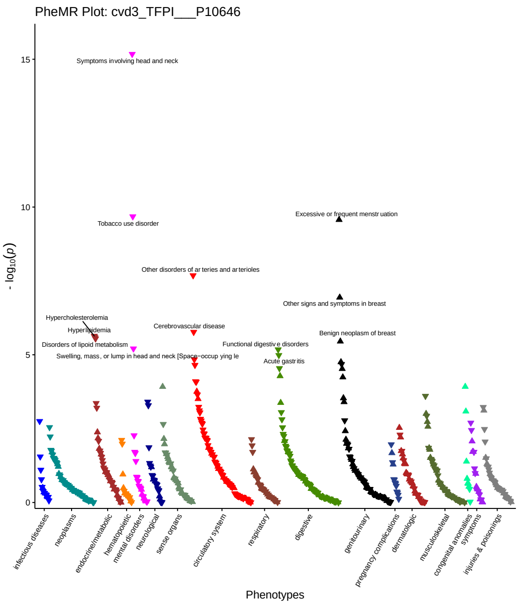


TMPRSS5

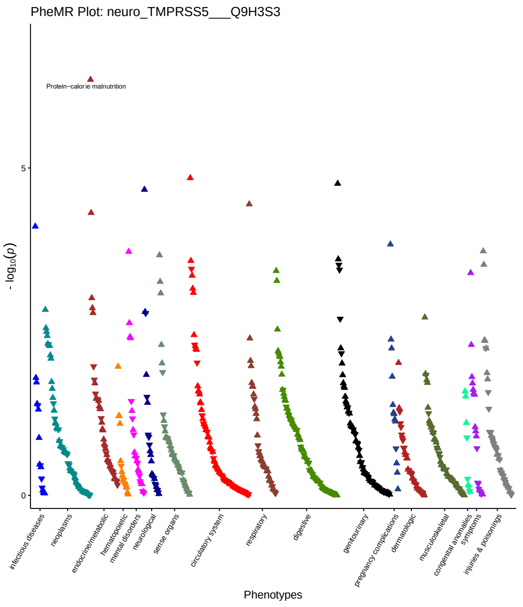


MMP12

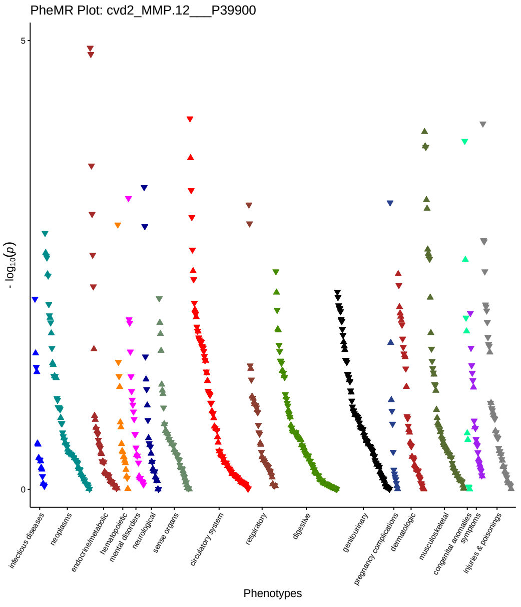


CD40

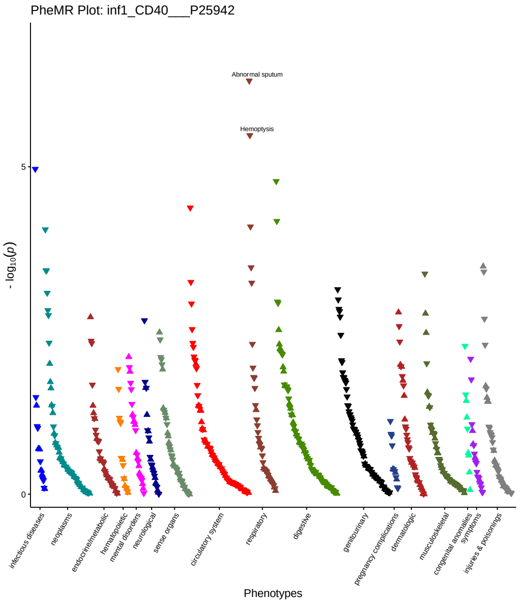

IL6RA CD6

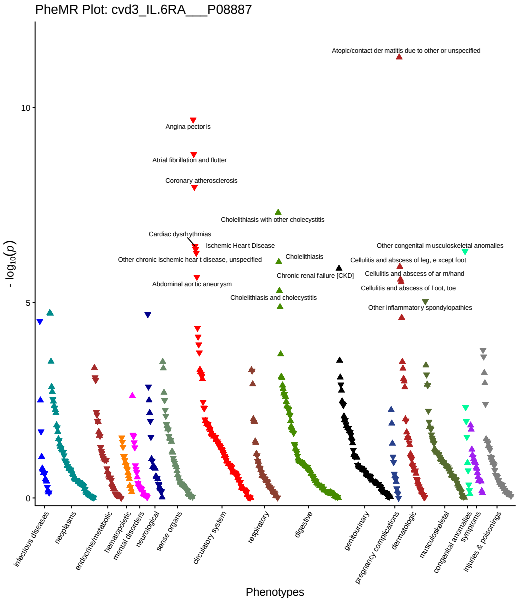


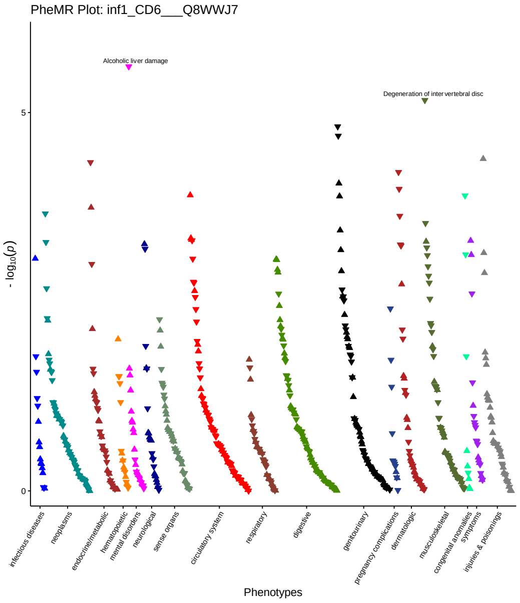


**Supplementary Figure 5.** Manhattan plots of the potential on-target side-effects from Phe-MR analysis.

Results can be perceived as the effects of per SD higher circulating protein level on each phenotype - triangle up represent risk-conferring effects and triangle down represents protective effects. If the effect direction of the target protein on the phenotype is consistent with that on stroke outcomes, it represents “beneficial” additional indications through intervention of circulating protein level. Conversely, opposing effect directions of the target protein on the phenotype and stroke represents “deleterious” side-effects. For example, a higher level of TFPI is associated with lower risk of ischemic stroke and so downface triangle represents “beneficial effects”, while the upward triangle represents “deleterious effects” when the hypothetical intervention increases TFPI levels. Associations above the pink line passed Bonferroni significance $P \leq 0.05/6/784 = 1.06\times10^{-5}$).

## CD40

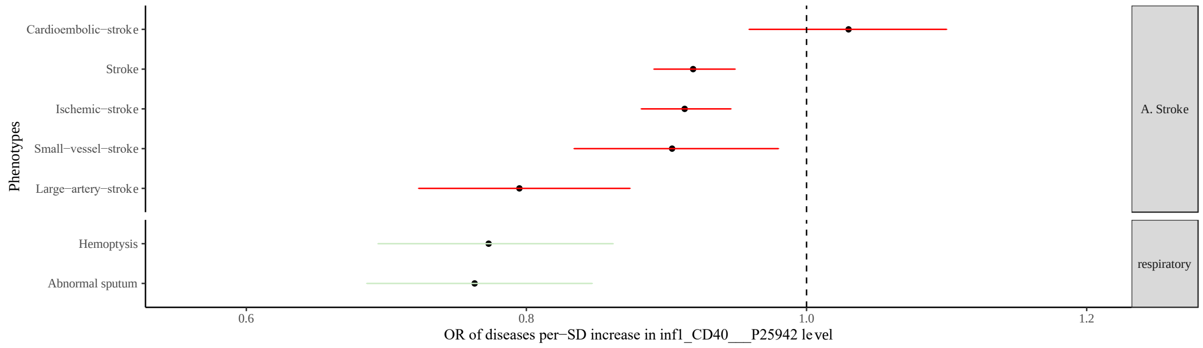


## CD6

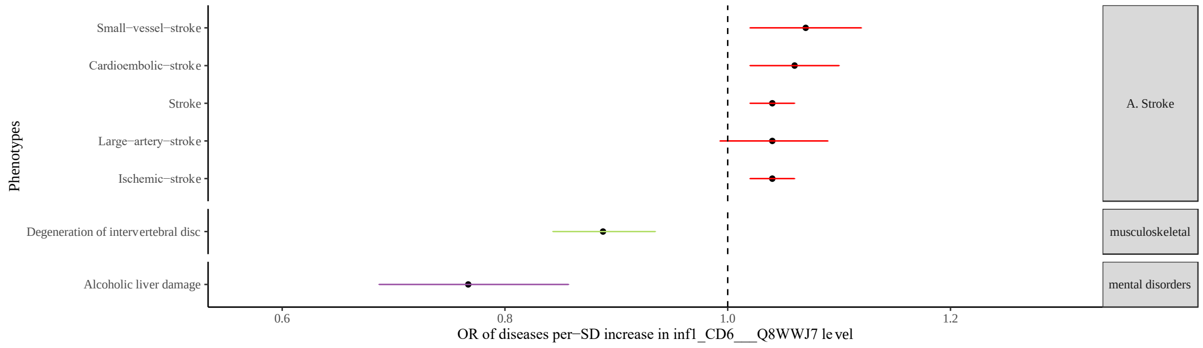


## MMP12

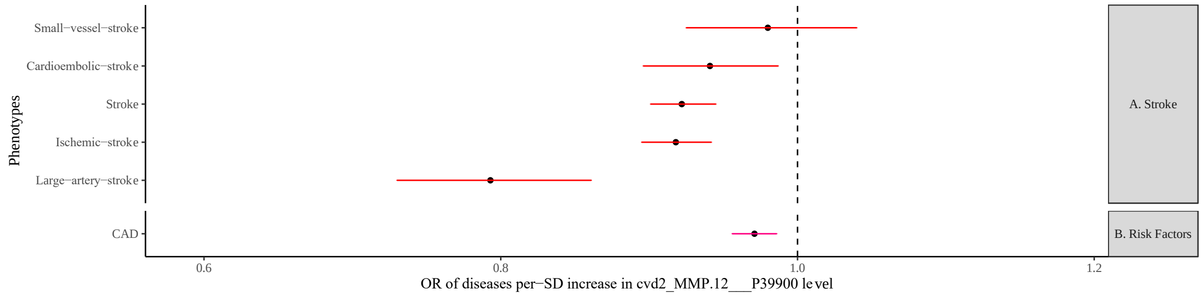

## IL6RA

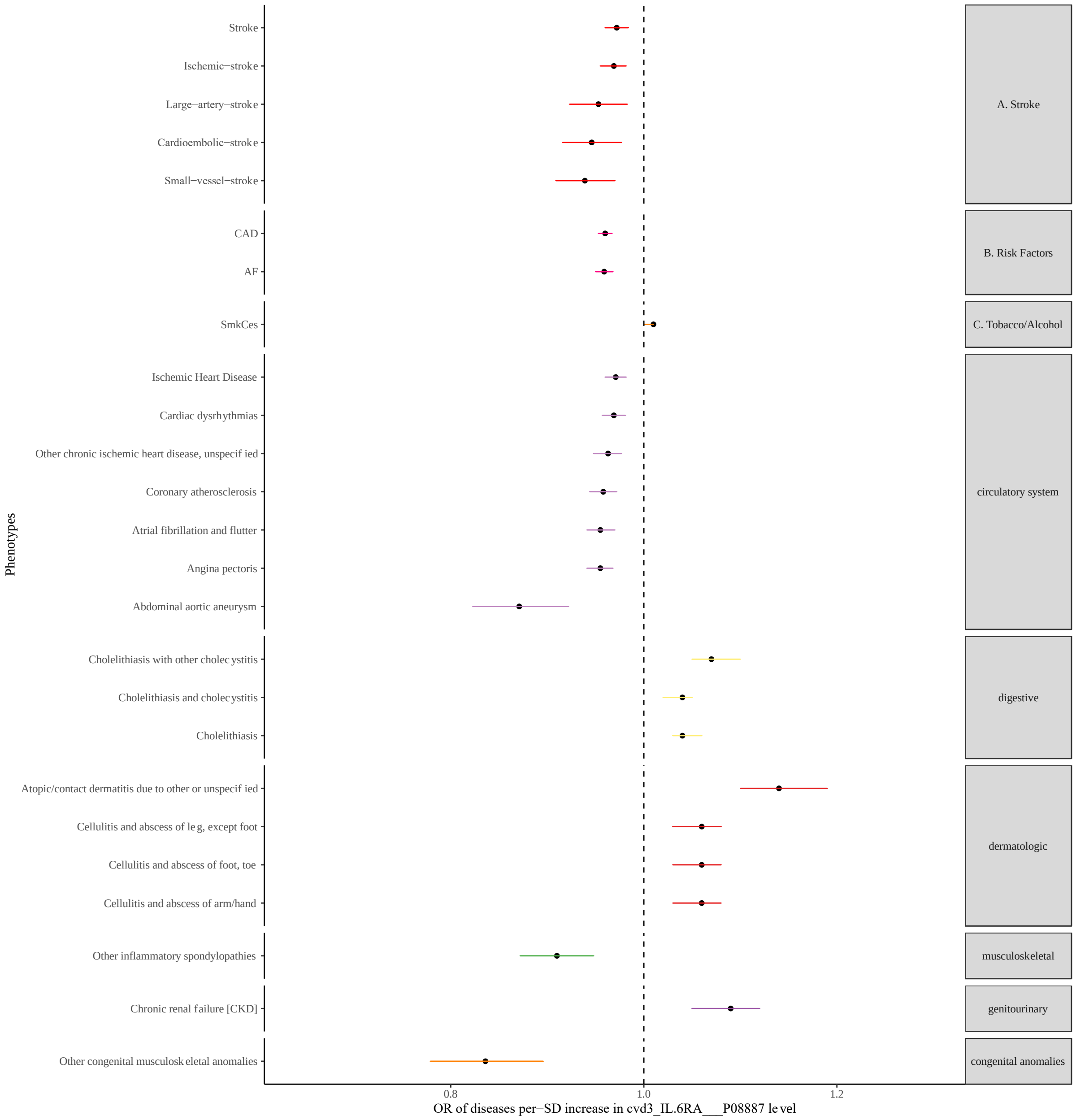


**Supplementary Figure 6.** Forest plots illustrating the potential on-target side-effects associated with causal proteins revealed by Phe-MR analysis.

Results can be perceived as the effects of per SD higher circulating protein level on each phenotype. If the effect direction of the target protein on the phenotype is consistent with that on stroke outcomes, it represents “beneficial” additional indications through intervention of circulating protein level. Conversely, opposing effect directions of the target protein on the phenotype and stroke represents “deleterious” side-effects. For example, a higher level of TFPI is associated with lower risk of ischemic stroke and so phenotypes with OR<1 represents “beneficial effects”, OR>1 represents “deleterious effects” when the hypothetical intervention increases TFPI levels. Only significant associations that passed Bonferroni correction ($P \leq 0.05/6/784 = 1.06\times10^{-5}$) were plotted. The dots are the causal estimates on the OR scale, and whiskers represent the 95% confidence intervals for these ORs.

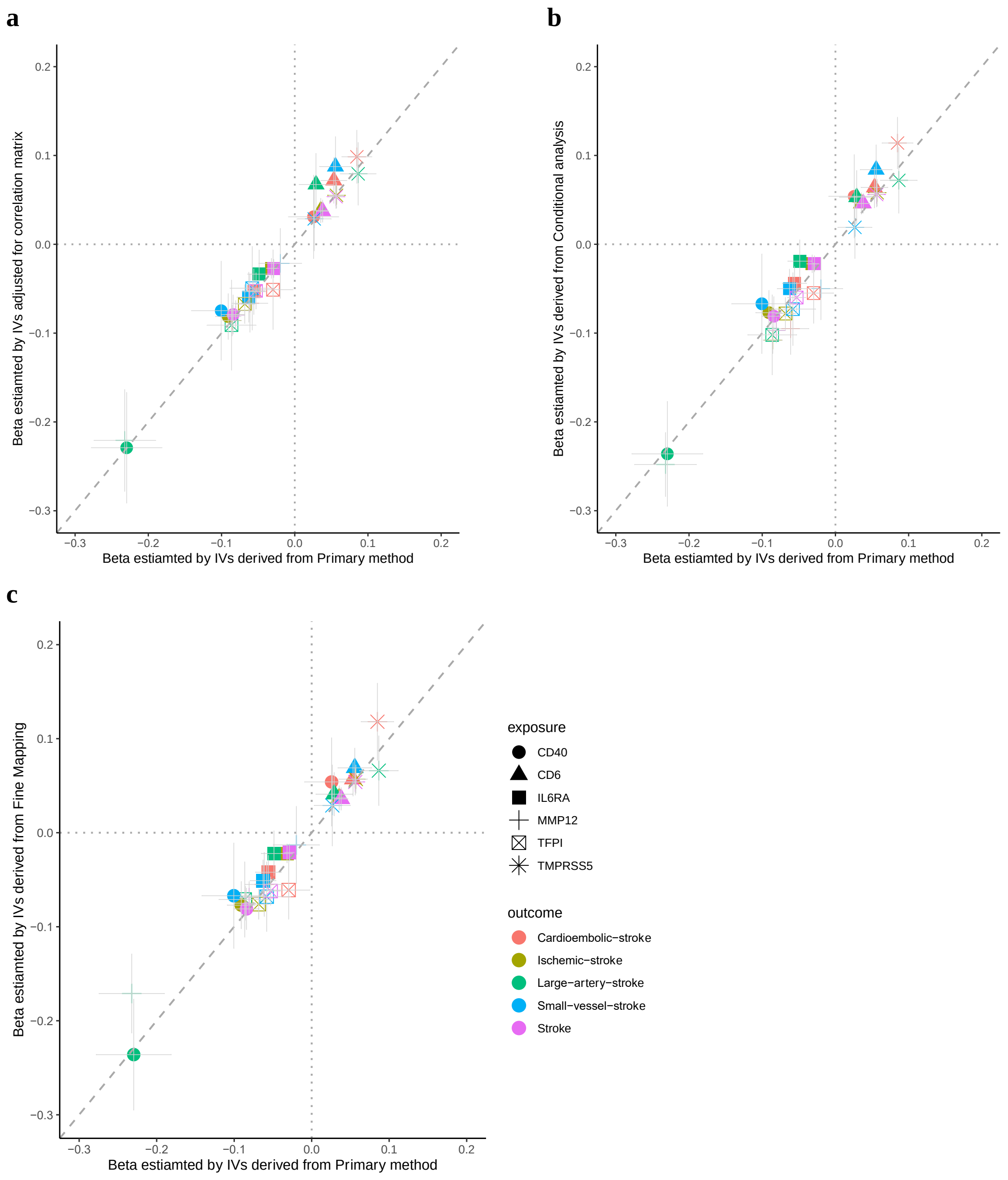


**Supplementary Figure 7.** Correlations of causal effect sizes of six proteins on stroke outcomes with IVs derived from primary method and others.

Primary method (IVs derived from LD clumping at $P_{pQTLs} < 5\times10^{-8}$ at $R^2 < 0.1$) versus IVs adjusted for correlation matrix (a); IVs derived from conditional analysis (b); and IVs derived from fine mapping (c). Scatter plots show the estimated effect sizes and 95% confidence intervals on proteins and risk of stroke outcomes, with each shape represents each protein and each colour represents each stroke outcome. The dashed grey line is a reference line representing y=x. Abbreviations: CD40 = B Cell Surface Antigen CD40; TFPI = Tissue Factor Pathway Inhibitor; MMP12 = Matrix Metallopeptidase 12; IL6RA = Interleukin 6 Receptor Subunit Alpha; TMPRSS5= Transmembrane Serine Protease 5; CD6 = T-Cell Differentiation Antigen CD6.
