# Supplementary material for: Systematic Mendelian randomization using the human plasma proteome to discover potential therapeutic targets for stroke
Source: Nat Commun. 2022 Oct 17;13:6143. doi: 10.1038/s41467-022-33675-1 (PMC9576777; doi:10.1038/s41467-022-33675-1)
Supplement: Supplementary file 3 — Description of Additional Supplementary Files [file 41467_2022_33675_MOESM3_ESM.pdf]

Description of Additional Supplementary Files

File Name: Supplementary Data 1
Description: **Description of Proteins assayed by Olink Platform**
Notes: Olink Protein ID: Olink assay protein ID, named as "OlinkPanel_HUGOGeneName___UniProtID". Gene coordinates (GRCh37): Genomic position in Build 37, showed as: "Chr:GeneStart-GeneEnd". IV Available: "TRUE" if at least one genome-wide significant signal (P<5e-08) from the protein GWAS is available; otherwise "FALSE". Duplicated: "TRUE" if the corresponding protein was also tested in other panel, otherwise "FALSE". Fail QC: "TRUE" if the corresponding protein failed quality control criteria in Olink assay; otherwise "FALSE". Available in Somalogic: "TRUE" if the corresponding protein was measured and passed QC in Somalogic INTERVAL (Sun et al, 2018); otherwise "FALSE". IV Available in Somalogic: "TRUE" if at least one genome-wide significant signal (P<5e-08) from the Somalogic INTERVAL (Sun et al, 2018) protein GWAS is available; otherwise "FALSE".

File Name: Supplementary Data 2
Description: **Instrumental variants for 15 stroke significant proteins (cis+trans) and annotations**
Notes: SNPID: RSID or "chr:position" in CRCh37 genome build. Gene: genes that in proximity to the SNP. SNP Func: SNP function of the corresponding gene. Allele effects on Exposure: the association summary statistics of the SNP on exposure. Allele effects on Outcome: the association summary statistics of the SNP on outcome. FDR: false discovery rate (adjusted p-value, uses the Benjamin & Hochberg ("BH") correction).

$$F = \frac{beta.exposure^2}{se.exposure^2}$$

$$R^2 = 2 \times MAF \times (1 - MAF) \times beta.exposure^2$$

File Name: Supplementary Data 3
Description: **MR results of proteome and stroke outcomes**
a) Proteins with only cis-pQTLs as instrumental variables.
b) Proteins with cis- and trans-pQTLs or only trans-pQTLs as instrumental variables.
Notes: Exposure: Plasma proteins; Outcomes - Stroke outcomes. N_SNP - number of instrumental variables (IVs). MR-IVW (Causal estimate derived from wald's ratio (N_SNP = 1) or from MR-IVW multiplicative fixed-effects model (N_SNP <= 3) or MR-IVW multiplicative random-effects model (N_SNP > 3). beta - causal effect size estimate. se - standard error (se). Pval - P value for causal estimate. MR-Egger (Causal estimate derived from MR-Egger method (N_SNP >= 3). MR-PRESSO (Causal estimate derived from MR-PRESSO with outlier-corrected (N_SNP >= 4). GlobalTest_Pval: P value of global pleiotropy derived from MR-PRESSO. MR-ConMix (Causal estimated derived from MR contamination mixture model (N_SNP >= 3). Beta (95% CI): causal estimate and its 95% confidential interval. Egger_intercept (Pval): Egger-Intercept and its P values for horizontal pleiotropy by MR-Egger. Q_statistics (Pval): Cochran Q statistics derived from IVW method and its P values for

heterogeneity. Heterogeneity_I^2: a measure of heterogeneity from MR-IVW. I2=(Q-df)/Q * 100%, if $I^2<0$, set it to 0. Q: Cochran Q statistics; df: degree-of-freedom.
NA: Not available/Not Appropriate. FDR: false discovery rate (adjusted p-value, uses the Benjamin & Hochberg ("BH") correction).

File Name: Supplementary Data 4
Description: **MR results of Proteome and Stroke, with IVs adjusted for correlation matrix**
Notes: Exposure: Plasma proteins; Outcomes - Stroke outcomes; N_SNP - number of instrumental variables (IVs). MR-IVW adjusted for IV correlation matrix (Causal estimate derived from from MR-IVW multiplicative fixed-effects model (N_SNP <= 3) or MR-IVW multiplicative random-effects model (N_SNP > 3). beta - causal effect size estimate; se - standard error (se); Pval - P value for causal estimate. Heterogeneity_I^2: a measure of heterogeneity from MR-IVW. I^2=(Q-df)/Q * 100%, if $I^2<0$, set it to 0. Q: Cochran Q statistics; df: degree-of-freedom.
NA: Not available/Not Appropriate.

File Name: Supplementary Data 5
Description: **Instrumental variables IV(s) derived from conditional analysis and fine-mapping for 6 stroke proteins**
Notes: IV_Method: statistical methods to derive IV(s). SNPID: named as rs id. chromosome: in CRCh37 genome build. position: in CRCh37 genome build. Allele effects on Exposure: the association summary statistics of the SNP on exposure. Allele effects on Outcome: the association summary statistics of the SNP on outcome.

$$F = \frac{beta.exposure^2}{se.exposure^2}$$

$$R^2 = 2 \times MAF \times (1 - MAF) \times beta.exposure^2$$

File Name: Supplementary Data 6
Description: **MR results of proteome and stroke outcomes using IV(s) that were derived from conditional analysis and fine-mapping (Supplementary Data 5)**
a) Proteins IV(s) derived from conditional analysis.
b) Proteins IV(s) derived from fine-mapping.
Notes: Exposure: Plasma proteins; Outcomes - Stroke outcomes; N_SNP - number of instrumental variables (IVs). MR-IVW (Causal estimate derived from wald's ratio (N_SNP = 1) or from MR-IVW multiplicative fixed-effects model (N_SNP <= 3) or MR-IVW multiplicative random-effects model (N_SNP > 3). beta - causal effect size estimate; se - standard error (se); Pval - P value for causal estimate. MR-Egger (Causal estimate derived from MR-Egger method (N_SNP >= 3). MR-PRESSO (Causal estimate derived from MR-PRESSO with outlier-corrected (N_SNP >= 4). GlobalTest_Pval: P value of global pleiotropy derived from MR-PRESSO. MR-ConMix (Causal estimated derived from MR contamination mixture model (N_SNP >= 3). Beta (95% CI): causal estimate and its 95% confidential interval. Egger_intercept (Pval): Egger-Intercept and its P values for horizontal pleiotropy by MR-Egger; Q_statistics (Pval): Cochran Q statistics derived from IVW method and its P values for heterogeneity. Heterogeneity_I^2: a measure of heterogeneity from MR-IVW. I^2=(Q-df)/Q * 100%, if $I^2<0$, set it to 0. Q: Cochran Q statistics; df: degree-of-freedom.

NA: Not available/Not Appropriate. *Data in bold showed statistically significant causal relationship between exposure and outcome. [* Significant: MR-IVW or MR-ConMix causal estimate Pval < 0.05/6 protein = 0.008].

File Name: Supplementary Data 7
Description: **Reverse MR results: Stroke as exposure and Proteome as outcome**
Notes: Exposure: Plasma proteins; Outcomes - Stroke outcomes; N_SNP - number of instrumental variables (IVs). MR-IVW (Causal estimate derived from wald's ratio (N_SNP = 1) or from MR-IVW multiplicative fixed-effects model (N_SNP <= 3) or MR-IVW multiplicative random-effects model (N_SNP > 3). beta - causal effect size estimate; se - standard error (se); Pval - P value for causal estimate. MR-Egger (Causal estimate derived from MR-Egger method (N_SNP >= 3). MR-PRESSO (Causal estimate derived from MR-PRESSO with outlier-corrected (N_SNP >= 4). GlobalTest_Pval: P value of global pleiotropy derived from MR-PRESSO. MR-ConMix (Causal estimated derived from MR contamination mixture model (N_SNP >= 3). Beta (95% CI): causal estimate and its 95% confidential interval. Egger_intercept (Pval): Egger-Intercept and its P values for horizontal pleiotropy by MR-Egger; Q_statistics (Pval): Cochran Q statistics derived from IVW method and its P values for heterogeneity. Heterogeneity_I^2: a measure of heterogeneity from MR-IVW. I^2=(Q-df)/Q * 100%, if I^2<0, set it to 0. Q: Cochran Q statistics; df: degree-of-freedom.
NA: Not available/Not Appropriate.

File Name: Supplementary Data 8
Description: **Colocalization results for stroke and/or stroke risk factors associated proteins.**
a) HyPrColoc results for 6 stroke-associated proteins. [Input traits for HyPrColoc: pQTLs + 5 stroke outcomes];
b) PwCoCo results: IL6RA pQTLs + stroke outcomes;
c) HyPrColoc results for all proteins that associated with Stroke and/or Stroke Risk Factors. [Input traits for HyPrColoc: pQTLs + 5 stroke outcomes + all stroke risk factors];
d) HyPrColoc results for 5 Stroke associated proteins. [Input traits for HyPrColoc: pQTLs + significant traits from PheMR results in Supplementary Data 15(a) for each corresponding protein].
Notes: Regional summary stats for HyPrColoc analysis: +/- 1MB window of genomic region that encoded the according protein. Prior probability for HyPrColoc method: uniform priors. PR* (regional probability threshold): PR* = 0.8. PA* (alignment probability threshold): PA* = 0.8. posterior_prob: The posterior probability of colocalization traits identified. Regional_prob: The probability of traits identified share an association region. candidate_snp: a single variant that is identified as a candidate shared variant. posterior_explained_by_snp: the proportion of posterior probability explained by the candidate SNP.

File Name: Supplementary Data 9
Description: **Genetic risk loci of stroke risk factors from corresponding GWASs**
a) Table of stroke risk factors.
b) Table of IV(s) of stroke risk factors.
Notes: Column name: TraitID: Risk Factor; SNPID: named as "chr:pos"; CHR: chromosome; POS(hg19): genomic position in hg19 genome build; rsID: SNP rs id; EA: effect allele; OA:

other allele; EAF: effect allele frequency; BETA: effect size/log (odds ratio); SE: standard error; PVAL: P value of association test.

File Name: Supplementary Data 10
Description: **MR results of risk factors versus stroke outcomes**
Notes: Exposure: Plasma proteins; Outcomes - Stroke outcomes; N_SNP - number of instrumental variables (IVs). MR-IVW (Causal estimate derived from wald's ratio (N_SNP = 1) or from MR-IVW multiplicative fixed-effects model (N_SNP <= 3) or MR-IVW multiplicative random-effects model (N_SNP > 3). beta - causal effect size estimate; se - standard error (se); Pval - P value for causal estimate. MR-Egger (Causal estimate derived from MR-Egger method (N_SNP >= 3). MR-PRESSO (Causal estimate derived from MR-PRESSO with outlier-corrected (N_SNP >= 4). GlobalTest_Pval: P value of global pleiotropy derived from MR-PRESSO. MR-ConMix (Causal estimated derived from MR contamination mixture model (N_SNP >= 3). Beta (95% CI): causal estimate and its 95% confidential interval. Egger_intercept (Pval): Egger-Intercept and its P values for horizontal pleiotropy by MR-Egger; Q_statistics (Pval): Cochran Q statistics derived from IVW method and its P values for heterogeneity. Heterogeneity_I^2: a measure of heterogeneity from MR-IVW. I^2=(Q-df)/Q * 100%, if I^2<0, set it to 0. Q: Cochran Q statistics; df: degree-of-freedom.
NA: Not available/Not Appropriate.

File Name: Supplementary Data 11
Description: **Significant MR results of proteome and stroke risk factors (secondary outcomes)**
a) MR results using IV(s) derived from cis-pQTLs.
b) Proteins with cis- and trans-pQTLs or only trans-pQTLs as instrumental variables.
Notes: Exposure: Plasma proteins; Outcomes - Stroke outcomes; N_SNP - number of instrumental variables (IVs). MR-IVW (Causal estimate derived from wald's ratio (N_SNP = 1) or from MR-IVW multiplicative fixed-effects model (N_SNP <= 3) or MR-IVW multiplicative random-effects model (N_SNP > 3). beta - causal effect size estimate; se - standard error (se); Pval - P value for causal estimate. MR-Egger (Causal estimate derived from MR-Egger method (N_SNP >= 3). MR-PRESSO (Causal estimate derived from MR-PRESSO with outlier-corrected (N_SNP >= 4). GlobalTest_Pval: P value of global pleiotropy derived from MR-PRESSO. MR-ConMix (Causal estimated derived from MR contamination mixture model (N_SNP >= 3). Beta (95% CI): causal estimate and its 95% confidential interval. Egger_intercept (Pval): Egger-Intercept and its P values for horizontal pleiotropy by MR-Egger; Q_statistics (Pval): Cochran Q statistics derived from IVW method and its P values for heterogeneity. Heterogeneity_I^2: a measure of heterogeneity from MR-IVW. I^2=(Q-df)/Q * 100%, if I^2<0, set it to 0. Q: Cochran Q statistics; df: degree-of-freedom.
NA: Not available/Not Appropriate. pQTL: pQTL within a +/-1MB window around the protein-coded gene region is defined as cis-pQTL; elsewhere in the genome is defined as trans-pQTL. Cis-only / trans-only indicated that there is only cis-pQTLs / trans-pQTLs available for the corresponding protein, while cis+trans indicated there are multiple significant pQTLs across the genome associated with the corresponding protein.

File Name: Supplementary Data 12
Description: **MR results of 6 stroke-associated proteins and stroke risk factors (secondary outcomes)**
Notes: Exposure: Plasma proteins; Outcomes - Stroke outcomes; N_SNP - number of instrumental variables (IVs). MR-IVW (Causal estimate derived from wald's ratio (N_SNP = 1) or from MR-IVW multiplicative fixed-effects model (N_SNP <= 3) or MR-IVW multiplicative random-effects model (N_SNP > 3). beta - causal effect size estimate; se - standard error (se); Pval - P value for causal estimate. MR-Egger (Causal estimate derived from MR-Egger method (N_SNP >= 3). MR-PRESSO (Causal estimate derived from MR-PRESSO with outlier-corrected (N_SNP >= 4). GlobalTest_Pval: P value of global pleiotropy derived from MR-PRESSO. MR-ConMix (Causal estimated derived from MR contamination mixture model (N_SNP >= 3). Beta (95% CI): causal estimate and its 95% confidential interval. Egger_intercept (Pval): Egger-Intercept and its P values for horizontal pleiotropy by MR-Egger; Q_statistics (Pval): Cochran Q statistics derived from IVW method and its P values for heterogeneity. Heterogeneity_I^2: a measure of heterogeneity from MR-IVW. I^2=(Q-df)/Q * 100%, if I^2<0, set it to 0. Q: Cochran Q statistics; df: degree-of-freedom.
NA: Not available/Not Appropriate.

File Name: Supplementary Data 13
Description: **MR mediation results for 3 protein targets on stroke outcomes via risk factors.**
* Total effect is the effect of exposure on outcome derived from Two-sample MR.

File Name: Supplementary Data 14
Description: **UK Biobank phenotypes for Phenome-wide MR analysis**

File Name: Supplementary Data 15
Description: **Phenome-wide MR results of proteome and UKBB phenotypes**
a) Summary of significant PheMR results with causal estimates P_IVW < 1.06e-05.
*The side-effect column showed the effect direction of the exposure on the outcome; if the effect direction is consistent with that of stroke, it is beneficial effect (green √); otherwise is adverse effect (red X).
b) Summary Table for PheMR results of 6 proteins and all phenotypes from UKBB.

File Name: Supplementary Data 16
Description: **Validation of stroke-associated proteins in SOMAlogic platform.**
a). Instrumental variables for two stroke associated proteins derived from SOMAlogic platform. IV(s) were selected by LD clumping (R^2=0.1, P=5e-08)
b). Comparison of MR results of two stroke-association proteins in Olink and SOMAlogic platform.
