# Supplementary material for: Systematic Mendelian randomization using the human plasma proteome to discover potential therapeutic targets for stroke
Source: Nat Commun. 2022 Oct 17;13:6143. doi: 10.1038/s41467-022-33675-1 (PMC9576777; doi:10.1038/s41467-022-33675-1)
Supplement: Supplementary file 5 — Reporting Summary [file 41467_2022_33675_MOESM5_ESM.pdf]

nature portfolio

Corresponding author(s): Joanna M. M. Howson

Last updated by author(s): Aug 25, 2022

Reporting Summary

Nature Portfolio wishes to improve the reproducibility of the work that we publish. This form provides structure for consistency and transparency in reporting. For further information on Nature Portfolio policies, see our Editorial Policies and the Editorial Policy Checklist.

Statistics

For all statistical analyses, confirm that the following items are present in the figure legend, table legend, main text, or Methods section.

n/a	Confirmed	
☐	☒	The exact sample size (n) for each experimental group/condition, given as a discrete number and unit of measurement
☐	☒	A statement on whether measurements were taken from distinct samples or whether the same sample was measured repeatedly
☐	☒	The statistical test(s) used AND whether they are one- or two-sided<br>*Only common tests should be described solely by name; describe more complex techniques in the Methods section.*
☐	☒	A description of all covariates tested
☐	☒	A description of any assumptions or corrections, such as tests of normality and adjustment for multiple comparisons
☐	☒	A full description of the statistical parameters including central tendency (e.g. means) or other basic estimates (e.g. regression coefficient) AND variation (e.g. standard deviation) or associated estimates of uncertainty (e.g. confidence intervals)
☐	☒	For null hypothesis testing, the test statistic (e.g. F, t, r) with confidence intervals, effect sizes, degrees of freedom and P value noted<br>*Give P values as exact values whenever suitable.*
☐	☒	For Bayesian analysis, information on the choice of priors and Markov chain Monte Carlo settings
☒	☐	For hierarchical and complex designs, identification of the appropriate level for tests and full reporting of outcomes
☐	☒	Estimates of effect sizes (e.g. Cohen's d, Pearson's r), indicating how they were calculated

Our web collection on statistics for biologists contains articles on many of the points above.

Software and code

Policy information about availability of computer code

Data collection	INTERVAL Olink proteome GWAS were derived from a subset of 4,994 blood donors with genetic data enrolled in the INTERVAL BioResource that were processed for proteomic profiling using Olink platform.
Data analysis	We used publicly available software (URLs are listed below).<br>Genotype imputation: PBWT imputation algorithm, https://github.com/richarddurbin/pbwt/<br>Proteome GWAS: SNPTEST v.2.5.2, https://www.well.ox.ac.uk/~gav/snptest/<br>LD clumping: PLINK 1.90, www.cog-genomics.org/plink/1.9/<br>Fine mapping: FINEMAP v1.4, http://www.christianbenner.com/<br>LD matrix calculation: LDstore v2.0, http://www.christianbenner.com/<br>Colocalization of pQTLs with stroke and risk factors: HyPrColoc R package version 1.0, https://doi.org/10.5281/zenodo.4293559<br>Mendelian Randomisation: R packages ‘TwoSampleMR’ version 0.4.22, ‘MendelianRandomization’ version 0.4.1, and “MRPRESSO” version 1.0.<br>We used R (version 3.5.1) extensively to analyze data and create plots.

For manuscripts utilizing custom algorithms or software that are central to the research but not yet described in published literature, software must be made available to editors and reviewers. We strongly encourage code deposition in a community repository (e.g. GitHub). See the Nature Portfolio guidelines for submitting code & software for further information.

Data

Policy information about availability of data

All manuscripts must include a data availability statement. This statement should provide the following information, where applicable:

- Accession codes, unique identifiers, or web links for publicly available datasets
- A description of any restrictions on data availability
- For clinical datasets or third party data, please ensure that the statement adheres to our policy

Part of the INTERVAL Olink proteome GWAS summary statistics have already been published as part of larger collaborative meta-analyses from the SCALLOP Consortium (Folkersen 2020; https://doi.org/10.5281/zenodo.2615265) and the others are the subject of forthcoming GWAS discovery manuscripts and will be made available upon publication. URLs for GWAS summary statistics used for Mendelian randomization and colocalization analyses are available as follows: stroke outcomes (Malik 2018; http://www.megastroke.org/index.html), blood pressure (Surendran 2020; https://app.box.com/s/1ev9iakptips70k8t4cm8j347if0ef2u), atrial fibrillation (Nielsen 2018; http://csg.sph.umich.edu/willer/public/afib2018), type 2 diabetes (Mahajan 2018; http://diagram-consortium.org/), white matter hyperintensity (Persyn 2020; http://cerebrovascularportal.org/informational/downloads), body mass index (Pulit 2019; https://doi.org/10.5281/zenodo.1251813), alcohol consumption and smoking behaviour (Liu 2019; https://genome.psych.umn.edu/index.php/GSCAN), UK Biobank SAIGE GWAS (Zhou 2018, https://www.leelabsg.org/resources). Table 1 provides further information on the genetic data resources. All other data that support the findings of this study are available from the corresponding author upon reasonable request.

Field-specific reporting

Please select the one below that is the best fit for your research. If you are not sure, read the appropriate sections before making your selection.

☒ Life sciences ☐ Behavioural & social sciences ☐ Ecological, evolutionary & environmental sciences

For a reference copy of the document with all sections, see nature.com/documents/nr-reporting-summary-flat.pdf

Life sciences study design

All studies must disclose on these points even when the disclosure is negative.

Sample size	We used 4,994 blood donors enrolled in the INTERVAL BioResource for proteome GWAS. The The sample size was determined by the maximum number of individuals enrolled in the INTERVAL with both genotype data and Olink proteomic profiling.
Data exclusions	GWAS exclusions: 1) Sex mismatches 2) low call rates 3) duplicate samples 4) heterozygosity >3 sd from mean 5) non-European ancestry 6) relatedness (pi_hat> 0.187).<br>Olink exclusions: "Flagged" samples (technical failures)
Replication	We compared MR results for the 6 protein targets with existing SOMAlogic pQTLs measured in ~3,000 participants from INTERVAL study (Sun et al 2018). We found four (of the six stroke-associated proteins) were available in SOMAlogic, i.e. IL6R, MMP12, TFPI, and TMPRSS5. Both IL6R and MMP12 were associated with stroke outcome(s) in the Somalogic data. However, there were no significant cis-pQTLs (P>5e-08) for either TFPI or TMPRSS5, and so a comparison of these proteins was not possible.
Randomization	Samples were randomized by sex and recruitment center for genotyping and proteome measurement.
Blinding	Assay measurements were performed blind to any phenotype information.

Reporting for specific materials, systems and methods

We require information from authors about some types of materials, experimental systems and methods used in many studies. Here, indicate whether each material, system or method listed is relevant to your study. If you are not sure if a list item applies to your research, read the appropriate section before selecting a response.

Materials & experimental systems

n/a	Involved in the study	
☒	☐	Antibodies
☒	☐	Eukaryotic cell lines
☒	☐	Palaeontology and archaeology
☒	☐	Animals and other organisms
☐	☒	Human research participants
☒	☐	Clinical data
☒	☐	Dual use research of concern

Methods

n/a	Involved in the study	
☒	☐	ChIP-seq
☒	☐	Flow cytometry
☒	☐	MRI-based neuroimaging

Human research participants

Policy information about studies involving human research participants

Population characteristics	A subset of 4,994 blood donors at mean age of 61 years (SD 6.7 years) enrolled in the INTERVAL BioResource. The population characteristics details are available in the original publication [Di Angelantonio, Emanuele, et al. The Lancet 390.10110 (2017): 2360-2371.]
Recruitment	The participants of this study are a subset of the INTERVAL study. The recruitment details are available in the original publication [Di Angelantonio, Emanuele, et al. The Lancet 390.10110 (2017): 2360-2371.]
Ethics oversight	The participants of this study are a subset of the INTERVAL study. The ethics details are available in the original publication [Di Angelantonio, Emanuele, et al. The Lancet 390.10110 (2017): 2360-2371.]

Note that full information on the approval of the study protocol must also be provided in the manuscript.
